# First Maltese record of *Stephanopachys quadricollis* (Marseul, 1879) (Coleoptera, Bostrichidae)

**DOI:** 10.3897/zookeys.606.8753

**Published:** 2016-07-21

**Authors:** David Mifsud, Gianluca Nardi

**Affiliations:** 1 Institute of Earth Systems, Division of Rural Sciences and Food Systems, University of Malta, Msida MSD 2080, Malta; 2 Centro Nazionale per lo Studio e la Conservazione della Biodiversità Forestale “Bosco Fontana”, Sede di Bosco Fontana – Corpo Forestale dello Stato, Strada Mantova 29, I-46045 Marmirolo (MN), Italy; 3 Università degli Studi di Roma “Sapienza”, Dipartimento di Biologia e Biotecnologie “Charles Darwin”, Via Alfonso Borelli 50, I-00161 Rome, Italy

**Keywords:** Bostrichidae, new record, new synonymy, Malta, Italy

## Abstract

Three specimens of *Stephanopachys quadricollis* (Marseul, 1878) were recently found in Malta in UV light traps and represent the first record of this species for this country. Although *Stephanopachys quadricollis* is native to the Mediterranean basin, it is not yet clear if these Maltese records are due to a natural population or to an interception. Distributional, nomenclatural and biological data on this species are summarized, and a new synonymy is established: *Stephanopachys quadricollis* (Marseul, 1879) = *Stephanopachys quadraticollis* Kocher, 1956, **syn. n.**

## Introduction

The Bostrichidae of the Maltese Islands are represented by ten species of which two are definitely aliens but their establishment is unclear (Nardi and Mifsud 2015). A recent capture of *Stephanopachys quadricollis* (Marseul, 1879) in Malta, discussed herein, is recorded for the first time from the Maltese Islands.

## Material and methods

The beetles were identified following the work of Borowski and Węgrzynowicz (2012). The authorship of *Stephanopachys quadricollis* is attribute to Marseul (1879) (see forward “Notes”). The distribution pattern is expressed also by a chorotype according to Vigna Taglianti et al. (1999). The systematic and botanic nomenclature follow The Plant List (2013). Possible interpolations are given in square brackets. The following abbreviations are used in the text: ex = specimen/s; leg. = legit or legerunt.

### Acronyms of specimen depositories

CDM
 D. Mifsud private collection, Malta 


CGN
 G. Nardi private collection, Cisterna di Latina (Latina), Italy 


CNBFVR
 Centro Nazionale per lo Studio e la Conservazione della Biodiversità Forestale “Bosco Fontana” di Verona, Sede di Bosco Fontana, Marmirolo (Mantua), Italy 


MCSG
 C. Mancini collection c/o Museo civico di Storia naturale, Genoa, Italy  (R. Poggi, pers. comm., 2016)

MZUF
 Museo di Storia Naturale, Sezione di Zoologia “La Specola”, Università degli Studi di Firenze, Florence 


## Taxonomy

### Stephanopachys quadricollis

Taxon classificationAnimaliaColeopteraBostrichidae

(Marseul, 1878)

#### Material examined.

ITALY, Liguria: Monte di [= Mount of] Portofino [(Genova)], 1910, [C.] Mancini leg., 1 ex (MCSG); M.te [= Monte di] Portofino [(Genova)], VI.1923, Dr. [A.] Andreini leg., 1 ex (MZUF); Zignago [(La Spezia)], 17.VII.1963, S. Failla leg., 1 ex (MZUF). Calabria: [Sila National Park,] Camigliatello [(Cosenza), 1300 m], 18.VI.1978, [F. Angelini leg.], 1 ex (MZUF, collection F. Angelini); Parco Nazionale della Sila [= Sila National Park] (Cosenza), Monte Altare, 1580 m, 39°25.205700'N 16°34.694312'E, 24.VII.2009, L. Spada, M. Bardiani, A. Biscaccianti & A. Campanaro leg., [direct collection, in a forest of Pinus nigra ssp. laricio Maire (A.B. Biscaccianti & L. Spada, pers. comm., 2016)] 1 ex (CNBFVR). MALTA, Ħal-Far [35°48.760020'N, 14°30.480840'E], 1.IX.–5.XI.2015, 3 ♀♀, in UV light traps situated in human habitation, D. Mifsud leg. (CDM, CGN).

#### Chorotype and distribution.

Mediterranean (cf. Borowski 2007, as *Stephanopachys quadricollis* (Fairmaire, 1878) [sic!], Borowski and Węgrzynowicz 2007, as *Stephanopachys quadricollis* (Fairmaire in Marseul 1878) [sic!], Borowski and Węgrzynowicz 2012, as *Stephanopachys quadricollis* (Fairmaire, 1878) [sic!]). This species is recorded from: Algeria, Croatia, France (Corsica included), Greece, Israel, Italy (Sicily included), Lebanon, Morocco, Portugal, Spain (Balearic Islands included), Syria, Tunisia, Asiatic Turkey and Ukraine (cf. Halperin and Damoiseau 1980, Nardi 2004, Borowski 2007, Brustel et al. 2013). Moreover, it was intercepted in Sweden (Lundberg 1995, Borowski 2007, Brustel et al. 2013), Germany and Argentina (Walker 2005), while the record for Israel (Halperin and Damoiseau 1980) was recently overlooked (Borowski 2007, Brustel et al. 2013).

#### Ecology.

*Stephanopachys* Waterhouse, 1888 is the sole genus of Bostrichidae specialized to feed on gymnosperms (Liu et al. 2008) and was considered as exclusively phloeophagous (cf. Lesne 1911, Lawrence 2010, Brustel et al. 2013). However, *Stephanopachys conicola* Fisher, 1950, a Nearctic species develops in cones of *Pinus monophylla* Torr. et Fr. and on decaying *Juniperus occidentalis* Hooker berries (Borowski and Węgrzynowicz 2012, Tonkel et al. 2014), whereas *Stephanopachys linearis* (Kugelann, 1792), a Palaearctic species, develop on coniferous trees (*Abies alba* Mill., *Pinus sylvestris* L., etc.) (Borowski and Węgrzynowicz 2012, Brustel et al. 2013), but exceptionally also in old wood of *Quercus* (Koch 1989).

*Stephanopachys quadricollis* is found from sea level up-to mountain biotopes (cf. Kocher 1956, as *Stephanopachys quadraticollis* [sic!], Angelini 1986, 1991, Bahillo de la Puebla et al. 2007, Brustel et al. 2013) and the most important host-plants include *Pinus halepensis* Mill. and *Pinus pinaster* Aiton (Perris 1862, as Dinoderus (Apate) substriatus Payk. [misidentification], Lesne 1897, Sahlberg 1913b, Español 1955, 1965, Halperin and Damoiseau 1980, Bahillo de la Puebla et al. 2007, Borowski and Węgrzynowicz 2012, Brustel et al. 2013). The species is however known to develop on other Pinaceae including: *Abies alba* (Lesne 1897), *Cedrus atlantica* (Endl.) Manetti ex Carrière, *Pinus nigra* J.F.Arnold and *Pinus sylvestris* (Brustel et al. 2013). The larvae of *Stephanopachys quadricollis* develop in or under bark of death wood in damaged trees which are still alive (under partially removed bark, in or near wounds following prunning by forest personel or lightning) (Brustel et al. 2013). The larvae of *Clanoptilus marginellus* (Olivier, 1790) (Coleoptera, Malachiidae) (Perris 1862, as *Malachius marginellus* Fab.), *Opilo domesticus* (Sturm, 1837) and *Opilo mollis* Linnaeus, 1758 (Coleoptera, Cleridae) are predatory on those of *Stephanopachys quadricollis* (Perris 1862, Lesne 1904), whereas *Entedon stephanopachi* Heqvist, 1959 (Hymenoptera, Eulophidae) is a primary parasitoid of other *Stephanopachys* species in Sweden and USA (Heqvist 1959, Schauff 1988, Gumovsky 2010).

#### Notes.

The Maltese specimens of *Stephanopachys quadricollis* do not show significant differences from those examined from other territories. In Italy, this species is rare and localized and was recorded from the following regions: Piemonte (Lesne 1897, Schilsky 1899, Lesne 1901, Luigioni 1929, Porta 1929), Liguria (Luigioni 1929), Basilicata (Angelini 1986), Calabria (Angelini 1991) and Sicily (Vitale 1928, Luigioni 1929, Porta 1929, Audisio et al. 1995, Sparacio 1997, Nardi 2004). A record from Maritime Alps (Porta 1929), probably refers to French sites near the Italian border (Lesne 1897: 339, 1901: 84). Specimens from Piemonte (without locality of collection) were originally collected and recorded by Baudi as *Stephanopachys substriatus* (Paykull, 1800) (Baudi 1873, 1890, in both cases as *Dinoderus substriatus* Payk.). Lesne (1897, 1901) examined material collected by Baudi from Piemonte and recorded the presence of only *Stephanopachys quadricollis* from the mentioned region. Thus, Baudi’s records from Piemonte could in reality belong to only *Stephanopachys quadricollis*, since this latter species was described (Marseul 1879) only after the first record of Baudi (1873). However, during the present study it was not possible to re-examine this material. The record of Luigioni (1929) for Liguria, is probably based on the above cited specimen collected in 1910, since A. Dodero from the Museo civico di Storia naturale (Genoa), previously sent many unpublished records from this region to his best friend, P. Luigioni (R. Poggi, pers. comm., 2016).

Most of the above-mentioned Italian literature records provide only the region of collection for *Stephanopachys quadricollis*, with the consequence that few precise locations are known. These include: Cugno Ruggeri, 1400–1500 m, on the Pollino Massif in Basilicata (Angelini 1986); Camigliatello, 1250–1300 m, on the Sila plateau in Calabria (Angelini 1991); contrada Tremonti near Messina (Vitale 1928), and Messina (Luigioni 1929) in Sicily. *Stephanopachys quadricollis* was collected from very few coastal regions in Italy. These include localities in Sicily and Mount of Portofino in Liguria (see material examined). In Sicily, Vitale (1927) collected a specimen of this species beating *Juglans regia* tree on the 1^st^ of April 1927.

The occurrence of *Stephanopachys quadricollis* in Malta, is not the sole insular record so far known; this species is also recorded from the Balearic Islands (cf. Bahillo de la Puebla et al. 2007), from Meleda Island in Croatia (Ganglbauer 1904, Vrydagh 1961) and from Lesbos Island in Greece (Sahlberg 1913a, 1913b). Moreover it is also known to occur in some mainland coastal areas: e.g. Mount of Portofino in northern Italy (see above), in southern France (Brustel and Aberlenc 2014), in the Iberian Peninsula (Español 1955, 1965, 1974, Bahillo de la Puebla et al. 2007, Baena and Zuzarte 2013, as *Stephanopachys quadricollis* (Fairmaire, 1878) [sic!]) and in Turkey (Vrydagh 1962).

The native status of *Stephanopachys quadricollis* in Malta is highly probable considering the fact that is a typical Mediterranean species and that its main host plant, *Pinus halepensis* is autochthonous (Haslam et al. 1977). However, an anthropic origin cannot be excluded. The location from where the Maltese specimens were collected is mainly an industrialized area with several pharmaceutical companies however some pine trees are also present. Interceptions of Palaearctic *Stephanopachys* species are known in other countries. These include *Stephanopachys quadricollis* in Sweden, Germany and Argentina (see above), *Stephanopachys substriatus* (Paykull, 1800) in Belgium (Coulon 1993) and Germany (Lucht 1987, Köhler and Klausnitzer 1998, Geis 2002), and a southern European unidentified species in USA (Haack and Cavey 2000, Haack 2006).

As reported above, the Maltese specimens were taken at UV light. The use of light traps is a useful method to capture Bostrichidae. In fact, four other species of this family were previously collected at light in Malta (Nardi and Mifsud 2015), while 17 species were collected during a large light-traps project in Israel (Chikatunov et al. 2006). However, in Israel *Stephanopachys quadricollis* was not collected in this project possibly due to the fact that its abundance was reported as “sporadic and rare” (Halperin and Damoiseau 1980: 48). In France, in a large artificial forest of *Pinus pinaster*, a single specimen of *Stephanopachys quadricollis* was collected by an emergence trap (Brin et al. 2011), and the capture of *Stephanopachys* spp. using intercept traps is rare (Brustel et al. 2013).

In the IUCN Red List of European saproxylic beetles, *Stephanopachys quadricollis* is classified as “Least concern” (Nieto and Alexander 2010), whereas in the Italian list its status is indicated as “Vulnerable” (Nardi et al. 2014, 2015). The only recent Italian record of this species is from the Sila National Park, and this provides further evidence of the coleopterological importance of this Park (cf. Angelini 1991, Mazzei et al. 2011), where the species probably develops on Pinus nigra ssp. laricio Maire. This same host plant for *Stephanopachys quadricollis* was also recorded by Sainte-Claire Deville (1914: 545) in Corsica. From a conservation point of view, *Stephanopachys quadricollis* is known from other Italian localities such as Pollino and Portofino which are already designated as protected areas and this should therefore contribute towards the survival of this species in Italy.

It must be underlined that Heyden (1891: 468) erroneously considered *Stephanopachys quadricollis* as a synonym of *Stephanopachys substriatus* (Paykull, 1800), and attributed the description of the former to “Frm.Ab’. 1879. 83. [= Fairmaire, 1879: 83, L’Abeille])”. Later, he (Heyden 1906: 421) citing Lesne (1897: 339), listed correctly *Stephanopachys* “*quadricollis* Mars. Ab’. 1879. 83 [= Marseul 1879: 83]” as a valid species. Fairmaire was indicated as the one who described this species as “(Fairmaire, 1878)” by various recent authors (Borowski 2007, Baena and Zuzarte 2013, Nardi et al. 2014, Borowski and Węgrzynowicz 2012, Nardi et al. 2015) or as “(Fairmaire in Marseul, 1878)” by others (Borowski and Węgrzynowicz 2007, Liu 2010). Nevertheless, this authorship is erroneous, since, this species was described by Marseul (1879: 83), as “*Dinoderus 4-collis* Fairm.”, who also wrote: “Discovered by M. Lamey, and described by M. Leon Fairmaire, I do not know where” [translated]. Probably, the material described by Marseul (1879) was labeled as “*Dinoderus 4-collis* Fairm.”, but this was an unpublished name. In fact, Bedel (1894: 149, footnote 3) wrote: “This species [= *Stephanopachys quadricollis* Mars.] was never described by Fairmaire; its first description appeared on *L’ Abeille*, XVIII, Nouv. [2], p. 83 (1878). I saw the *type* [see also Vrydagh (1962: 6)] of S. de Marseul, belonging to M. A. Lamey” [translated]. This opinion was followed by almost all other authors (e.g. Lesne 1897, Schilsky 1899, Lesne 1901, Ganglbauer 1904, Lesne 1904, 1905, Heyden 1906, Lesne 1909, Sainte-Claire Deville 1910, 1914, Corrêa De Barros 1924, Winkler 1927, Luigioni 1929, Porta 1929, Portevin 1931, Peyerimhoff 1933, Normand 1936, Sainte-Claire Deville 1937, Lesne 1938, 1939, Seabra 1943, as *Stephanopachys quadricollis* Marsh. [sic!], Novak 1952, Español 1955, Kocher 1956, Vrydagh 1956, 1960, 1961, 1962, Español 1965, 1974, Halperin and Damoiseau 1980, Lucht 1987, Audisio et al. 1995, Lundberg 1995, Sparacio 1997, Nardi 2004, Walker 2005, Bahillo de la Puebla et al. 2007, Brin et al. 2011, Brustel et al. 2013, Brustel 2014, Brustel and Aberlenc 2014), nevertheless as stated by Heyden (1891, 1906) and by other authors (López-Colón 2000, López-Colón et. al. 2001, Grosso-Silva 2005), the year when *Stephanopachys quadricollis* was described is 1879 and not 1878. The year 1878 was included in the title of number 21 of the section “Nouvelles et Faits divers de L’Abeille” that includes the original description of this species (Marseul 1879). Numbers 18–25 of this section belong to volume 17 of 1879 and this information is also reported in the “Tables of Contents” [translated] of the same volume.

Finally, Kocher (1956: 114) published an unjustified emendation (ICZN 1999, art. 33.2.1) of *Stephanopachys quadricollis*: “*Stephanopachys quadraticollis* (err. *quadricollis*) Mars.”. Unfortunately, this name is an available one (ICZN 1999, art. 19.1) and so the following synonymy is here established: *Stephanopachys quadricollis* (Marseul, 1879: 83) = *Stephanopachys quadraticollis* Kocher, 1956: 114, **syn. n.**

## Supplementary Material

XML Treatment for Stephanopachys quadricollis
